# Development and evaluation of floating alginate microspheres for oral delivery of anthocyanins – A preliminary investigation

**DOI:** 10.1002/fsn3.451

**Published:** 2016-12-21

**Authors:** Giovana B. Celli, Amyl Ghanem, Marianne S. Brooks

**Affiliations:** ^1^ Department of Process Engineering and Applied ScienceDalhousie UniversityHalifaxCanada

**Keywords:** anthocyanin, encapsulation efficiency, gastroretentive system, low density, release

## Abstract

The goal of this study was to develop floating microspheres that could be used as gastroretentive systems for the delivery of anthocyanins (ACNs). These compounds are absorbed in the stomach and small intestine, and insufficient residence time in these organs could result in limited absorption and contribute to degradation. The microparticles containing freeze‐dried haskap berry extract (321.96 ± 8.35 mg cyanidin 3‐glucoside equivalents per g) were prepared by ionotropic gelation of alginate (9%, w/w) with calcium ions (CaCl_2_ at 2%, w/v) in the gelation bath, with calcium carbonate as the gas‐generating compound (added at different ratios in the alginate/extract mixture). The effect of acetic acid concentration (2 and 10%, v/v) in the gelation medium was investigated. Increasing the carbonate : alginate weigh ratio from 0 to 3:4 resulted in different degrees of floatability, larger particles, higher encapsulation efficiency, and lower amount of ACN released. The power law equation fitted the experimental data well, indicating that release occurred mainly by diffusion. This is the first time floating microspheres are proposed as gastroretentive platforms for the delivery of ACNs.

## 1 Introduction

Gastroretentive systems (GRS) differ from conventional oral delivery platforms as they remain in the stomach is longer (Joseph, Lakshmi, & Jayakrishnan, 2002). For instance, Ma et al. (2008) showed that floating particles remained in the stomach of volunteers for over 5 hr compared to a residence time of 2.5 hr for nonfloating systems. These systems can increase the residence time of bioactive compounds that are stable under acidic conditions and absorbed in the stomach, such as anthocyanins (ACNs), a group of hydrophilic pigments (Delgado‐Vargas, Jiménez, & Paredes‐López, 2000) with health‐promoting properties (Cassidy et al., 2013; Wallace, 2011). These compounds have been associated with the reduced risk of degenerative diseases (Cassidy et al., 2013; Pascual‐Teresa, Moreno, & García‐Viguera, 2010; Wang & Stoner, 2008), including type 2 diabetes (van Dam, Naidoo, & Landberg, 2013; Guo & Ling, 2015).

Amongst the known sources of these compounds, haskap berries (*Lonicera caerulea* L.) exhibit a high concentration of ACNs in addition to other bioactive molecules (Celli, Ghanem, & Brooks, 2014), which makes them an interesting raw material for the development of value‐added products. These berries are native to Siberia and north‐eastern Asia (Bors et al., 2012), and have recently been introduced in the North American market.

Although the stomach is not often considered an absorption site, researchers have shown that ACNs are absorbed in this organ and in the initial portion of the small intestine (Celli, Ghanem, & Brooks, 2016a; Passamonti, Vrhovsek, Vanzo, & Mattivi, 2003), which could explain the rapid appearance of these compounds in the circulation (within 30 min after administration) (Vanzo, Vrhovsek, Tramer, Mattivi, & Passamonti, 2011). In general, ACNs are stable in the stomach (pH < 2) (Liang et al., 2012) and likely found in the stable flavylium cation form (Brouillard & Dubois, 1977). This ion has limited chances of being absorbed by passive diffusion (Lipinski, Lombardo, Dominy, & Feeney, 1997), and bilitranslocase found in the gastric mucosa has been suggested as the carrier responsible for its absorption (Passamonti et al., 2003). This protein is primarily involved in the hepatic detoxification process and transport of organic anions from the circulation into the hepatocytes (Battiston, Macagno, Passamonti, Micali, & Sottocasa, 1999). However, under certain conditions, bilitranslocase can reach saturation, that is, an increase in the amount of ACNs administered does not result in a proportionally higher bioavailability, as demonstrated by Adisakwattana, Yibchok‐Anun, Charoenlertkul, and Wongsasiripat (2011) and Keane et al. (2016). The ACNs that are not absorbed in the upper gastrointestinal (GI) tract are subject to extensive metabolism (Czank et al., 2013) and degradation (Liu et al., 2014).

The objective of the present study was to develop floating microparticles for the oral delivery of ACNs extracted from haskap berries, using an ionotropic gelation method with sodium alginate, calcium chloride, and calcium carbonate (CaCO_3_) as the hydrocolloid, crosslinking ion, and gas‐generating compound, respectively. CaCO_3_ releases carbon dioxide when reacted with acid, which permeates the alginate gel matrix resulting in the formation of bubbles or pores (Choi, Park, Hwang, & Park, 2002), allowing the particles to float. Different CaCO_3_:alginate weight ratios (0, 1:4, 1:2, and 3:4) were investigated and their effects on the encapsulate properties (particle size, encapsulation efficiency, and floating capacity) and release profiles were assessed in comparison to the control (nonfloating particles without CaCO_3_).

## 2 Material and Methods

### 2.1 Materials

Formic, acetic, and hydrochloric acids, reagent ethanol, calcium carbonate, potassium chloride, sodium acetate anhydrous, Tween^®^ 80, and sodium citrate were purchased from Fisher Scientific (Ottawa, ON, Canada). Calcium chloride 96% anhydrous and calcium carbonate 98% pure were purchased from Acros Organics (Fairlawn, NJ, USA). Low‐viscosity sodium alginate Protanal LFR5/60 (MW = 40 kDa, 65–75% guluronic acid) was kindly donated by FMC BioPolymer (Philadelphia, PA, USA).

### 2.2 Plant material

Frozen haskap berries (var. *Indigo Gem*) harvested at the Northern Light Orchards (Saskatchewan, Canada) were used in this study. The berries were halved and freeze‐dried in a Labconco FreeZone 4.5 L Bench‐top Freeze Dry System (Labconco, Kansas City, MO, USA) (final moisture content <5%, w/w). The freeze‐dried berries were stored in a desiccator at −18°C until extraction. Five ACNs were identified in this haskap berry variety, namely cyanidin 3,5‐diglucoside, cyanidin 3‐glucoside, cyanidin 3‐rutinoside, pelargonidin 3‐glucoside, and peonidin 3‐glucoside (Celli, Ghanem, & Brooks, 2015a). The total anthocyanin content was found to be 321.96 ± 8.35 mg cyanidin 3‐glucoside equivalents per g (Celli, Brooks, & Ghanem, 2016b).

### 2.3 Preparation of anthocyanin‐rich freeze‐dried haskap extract

The extraction of ACNs from freeze‐dried haskap berries and preparation of freeze‐dried extract was conducted according to Celli, Ghanem, and Brooks (2015b) and Celli et al. (2016b). Briefly, freeze‐dried samples were extracted under magnetic stirring (600 rpm, 10 min at room temperature) with 80% ethanol (acidified with 0.5% formic acid) in a solid to solvent ratio of 1:25 (w/v). The filtered extract was kept at −18°C until further use.

A rotary evaporator model RE‐51 (Yamato Scientific America Inc., Santa Clara, CA, USA) equipped with equipped with a vacuum pump V‐700 (Büchi Labortechnik AG, Flawal, Switzerland) and a water circulation cooler WKL 230 (Lauda, Lauda‐Königshofen, Germany) was used to remove the organic solvent prior to the fractionation (temperature was kept below 50°C). The fractionation of the evaporated extract in a C18 column was performed according to Kalt et al. (2008) with some modifications. Briefly, the evaporated extract was added to a prewashed column and washed with approximately two volumes of water. The bound components were eluted with 1.5 volume of reagent ethanol (acidified with 0.5% formic acid), followed by rotary evaporation and freeze‐drying. This procedure allowed the purification and concentration of ACNs in the freeze‐dried powder by 22‐fold. The powder was stored at −18°C until further use.

### 2.4 Preparation of calcium‐alginate microparticles

Microparticles with or without CaCO_3_ were prepared according to the procedure by Celli et al. (2015b) with some modification. The freeze‐dried extract was dissolved in distilled water at a concentration of 2 mg mL^−1^ under magnetic stirring. The pH was adjusted to approximately 4.8 with sodium citrate to prevent the gelation of alginate and precipitation as alginic acid. Then, sodium alginate was dissolved in this solution (9.0%, w/w) and stirred thoroughly for at least 2 hr. For the preparation of floating particles, CaCO_3_ was added to the alginate solution at different weight ratios (CaCO_3_:alginate = 1:4, 1:2, and 3:4, w/w), according to Choi et al. (2002). The gelation medium consisted of CaCl_2_ (2.0%, w/v) in 2% (v/v) or 10% (v/v) glacial acetic acid. The alginate solution was then extruded through a 26 G needle using a Fisher Syringe Pump Model 78‐0100I (Fisher Scientific, Holliston, MA, USA) at an average flow rate of 1.0 mL min^−1^ into 30 mL of gelation medium that was gently stirred (60 rpm). The distance between the tip of the needle and the gelation medium surface was fixed at 10 cm. The gelation was allowed to proceed for 10 min under stirring, after which the particles were recovered by vacuum filtration and washed with distilled water. Both the formation of the microspheres and the gelation were carried out at room temperature. Particles were dried at room temperature in the absence of light until constant weight was observed.

### 2.5 Particle characterization

#### 2.5.1 Determination of particle size

The particle size was determined using a caliper ruler. As the particles were not completely spherical, the size of the fresh and dry particles was determined by measuring the major axis of individual particles and reporting the average measurement from 10 particles. Wet and dry particles were measured to determine the degree of shrinkage after drying.

#### 2.5.2 In vitro floating capacity

The in vitro floating capacity was assessed by the visual observation method described by El‐Gibaly (2002), slightly modified, so that the percentage of particles that floated and the duration of buoyancy could be determined. Using a water bath shaker Model 2870 (Thermo Scientific), a fixed amount of individual microspheres (20) was placed into 100 ml of enzyme‐free simulated gastric fluid (0.1 N HCl containing 0.02% w/v Tween^®^ 80) and maintained at 37 ± 0.5°C, undisturbed. The floating lag time was determined by measuring the time taken for the encapsulates to rise to the surface and float. Both the percentage of floating particles (counted visually) and the duration of buoyancy (time during which the particles remained buoyant on the simulated gastric solution) (*n *=* *3) were determined at fixed time intervals during an 8‐hr period.

#### 2.5.3 Determination of anthocyanin encapsulation efficiency

Encapsulation efficiency (EE, %) was indirectly assessed by measuring the anthocyanin content in the recovered CaCl_2_ solution (i.e. amount not entrapped) by the pH differential method (Giusti & Wrolstad, 2001). Absorbances were measured at *λ*_*max*_ (determined as 515 nm) and 700 nm in a Genesys 10S UV‐Vis spectrophotometer (Thermo Scientific, Madison, WI, USA) against distilled water (blank).

#### 2.5.4 In vitro anthocyanin release

The release of ACNs from the floating particles was determined by the method described by Santos, Albarelli, Beppu, and Meireles (2013), with modifications. A known weight of particles (100 mg) was added to 150 mL of enzyme‐free simulated gastric fluid (0.1 N HCl containing 0.02% w/v Tween^®^ 80) maintained at 37 ± 0.5°C in a reciprocal shaker bath Model 2870 (Thermo Scientific), agitated at 95 cycles per minute. At specific time intervals, an aliquot of 5 mL was removed and replaced by the same volume of fresh media (at 37 ± 0.5°C). Samples were then filtered through a 0.45 μm syringe filter and analyzed spectrophotometrically at λ_*max*_ and 700 nm against a blank (distilled water) and a known concentration of ACNs in simulated gastric fluid.

ACN release kinetics were analyzed by plotting the mean release data (%) versus time (hr). In order to investigate the release mechanism, data were initially fitted to first‐order kinetics, Higuchi law, and Peppas power law equations. However, due to the considerably low regression coefficients (*R* ^*2*^), first‐order kinetics and Higuchi law were not considered for further analysis. Curves were fitted for the Peppas (or power law) equation (Ritger & Peppas, 1987; Siepmann & Peppas, 2001), using SigmaPlot™ version 13.0 software (Systat Software, Inc., Sao Jose, CA, USA), as follows:

(1) $$\frac{M_{t}}{M_{\infty }}=kt^{n},$$

where *M*_*t*_ and *M*_*∞*_ are the cumulative absolute amount of drug released at time *t* and infinite time, respectively; *k* is the kinetic constant; and *n* is the release exponent that characterizes the diffusional mechanism.

The difference in release profiles with the addition of CaCO_3_ was assessed by the similarity (*f*_*2*_) and difference (*f*_*1*_) factors (Moore & Flanner, 1996) calculated, using the following equations:

(2) $$f_{2}=50\cdot \log\left\{{\left[1+\left(\frac{1}{P}\right)\sum_{i=1}^{p}{\left(R_{i}-T_{i}\right)}^{2}\right]}^{-1/2}\cdot 100\right\}$$

(3) $$f_{1}=\left\{\left[\sum_{i=1}^{P}|R_{i}-T_{i}|\right]/\sum_{i=1}^{P}R_{i}\right\}\cdot 100,$$

where *P* is the number of time points, and *R*_*i*_ and *T*_*i*_ are the release value of the reference and test at time *i*, respectively. The release profiles were considered significantly different if *f*_*2*_ < 50. The *f*_*1*_ increases proportionally with the dissimilarity between the curves.

### 2.6 Statistical analysis

All the experiments were conducted in triplicate unless otherwise stated and results were reported as mean ± standard deviation. Data were analyzed by one‐way analysis of variance (ANOVA), using SPSS^®^ version 22 software (IBM Corp., Armonk, NY, USA). Tukey's test (*p *=* *.05) was used to assess significant differences between means.

## 3 Results

### 3.1 Determination of particle size

**Figure 1:**
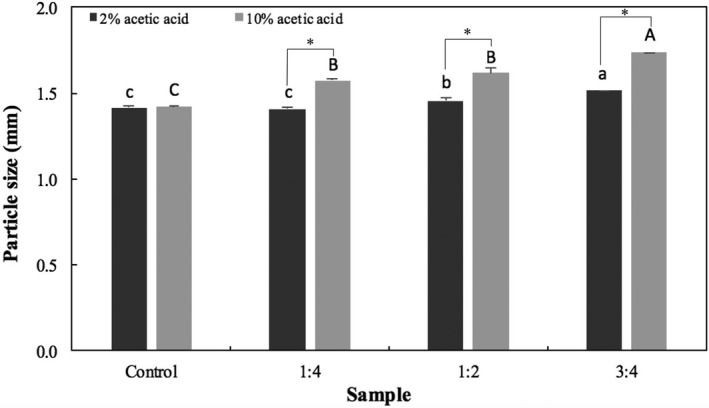
Particle size of dry microspheres prepared with different CaCO_3_:alginate ratios (1:4, 1:2, and 3:4) in comparison to the control (no carbonate added). Bars represent mean ± standard deviation (*n *=* *3). Those identified with the same lower‐ and uppercase letters are not significantly different within groups (*p *<* *.05, Tukey's test) for gelation media containing 2 and 10% acetic acid glacial, respectively. Stars (*) indicate significant difference (*p *<* *.05) between groups

Different CaCO_3_:alginate weight ratios were investigated for the preparation of floating particles and comparison with nonfloating (control) microspheres. Figure 1 shows that increasing the ratio of CaCO_3_ significantly increased the size of the microspheres in comparison to the control (with the exception of 1:4 ratio prepared with 2% acetic acid), in agreement with the results found by Choi et al. (2002). The use of different acetic acid concentrations also impacted particle size: microspheres extruded into 10% acetic acid solution were significantly (*p *<* *.05) larger than the ones prepared with 2% acetic acid.

### 3.2 In vitro floating capacity

**Figure 2:**
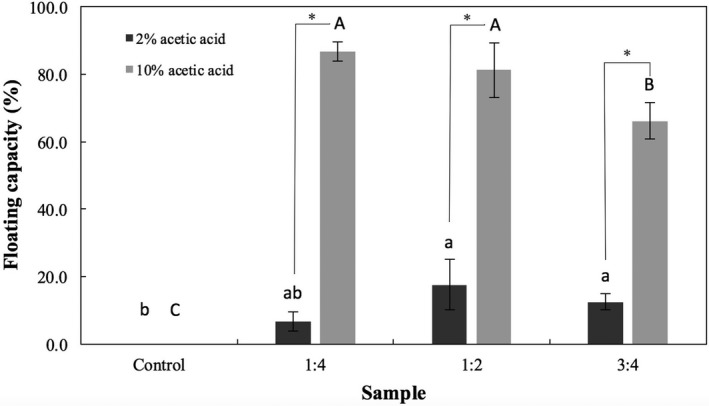
Floating capacity (i.e., overall percentage of microspheres that floated) of dry microspheres prepared with different CaCO_3_:alginate ratios (1:4, 1:2, and 3:4) in comparison to the control. Bars represent mean ± standard deviation (*n *=* *3). Those identified with the same lower‐ and uppercase letters are not significantly different within groups (*p *<* *.05, Tukey's test) for gelation media containing 2 and 10% acetic acid glacial, respectively. Stars (*) indicate significant difference (*p *<* *.05) between groups

The floating capacity is an important property of the microspheres, which would enable them to float on the gastric fluid and remain buoyant above the gastric content, increasing their residence time in the stomach. The floating capacity of the dry microspheres was evaluated in enzyme‐free simulated gastric fluid. Figure 2 presents the overall percentage of beads that floated during the duration of the experiment (8 hr). The control microspheres sank uniformly in the simulated fluid and were statistically different (*p *<* *.05) than the other samples. Particles containing CaCO_3_ prepared with 10% acetic acid floated immediately when placed in a simulated gastric fluid, whereas those prepared with a lower concentration of acid floated after variable periods (different lag times). Microspheres that floated in the simulated gastric fluid remained buoyant over the duration of the experiment.

### 3.3 Determination of anthocyanin encapsulation efficiency

**Figure 3:**
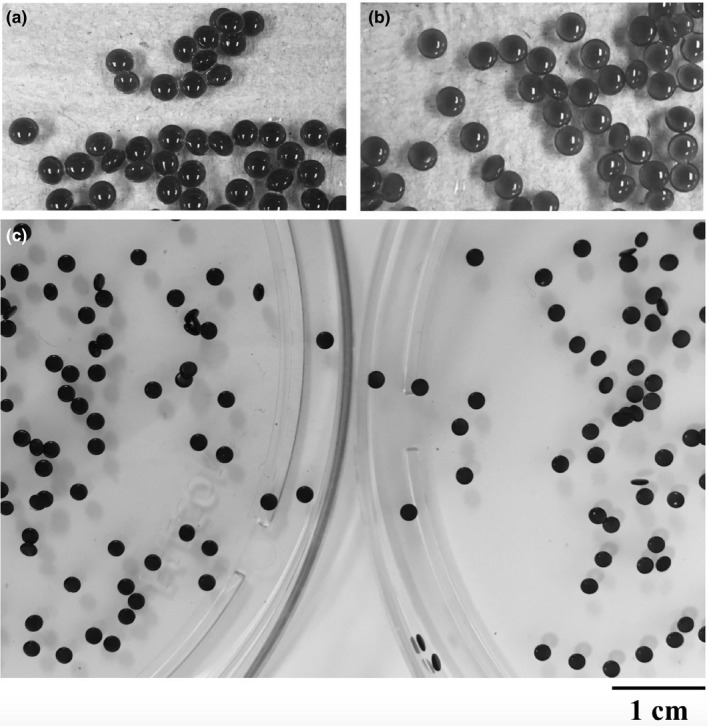
Visual observation of fresh nonfloating microspheres prepared with (a) 2% and (b) 10% acetic acid, and (c) after drying (2% ‐ left, 10% right) at room temperature shows the difference in EE, as indicated by the color of the particles

Taking into consideration the color intensity of the particles produced, the macroscopic appearance of the particles suggested that those prepared with 10% acetic acid had lower EE (i.e. the red color is less intense than the microspheres prepared with 2% acetic acid) (Figure 3). This preliminary observation was confirmed by spectrophotometric measurement: the EE of the control particles was 61.42 ± 1.90% and 57.04 ± 3.66% for samples prepared with 2 and 10% acetic acid, respectively.

**Figure 4:**
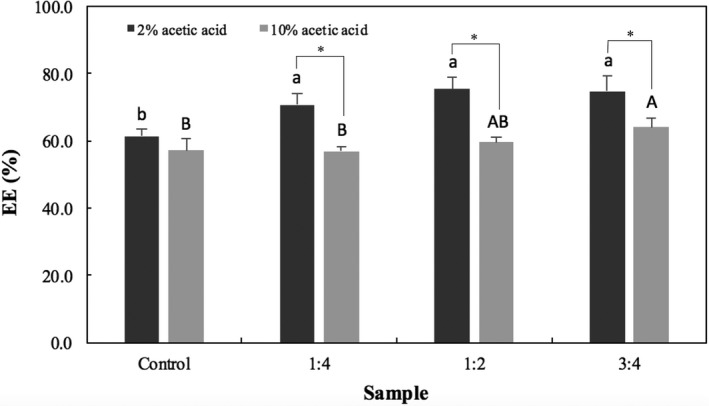
EE of microspheres prepared with different CaCO_3_:alginate ratios (1:4, 1:2, and 3:4) in comparison to the control (no carbonate added). Bars represent mean ± standard deviation (*n *=* *3). Those identified with the same lower‐ and uppercase letters are not significantly different within groups (*p *<* *.05, Tukey's test) for gelation media containing 2 and 10% acetic acid glacial, respectively. Stars (*) indicate significant difference (*p *<* *.05) between groups

Unlike previous reports (Shishu, Gupta, & Aggarwal, 2007), the addition of CaCO_3_ significantly increased the EE, especially for the microspheres gelled in cross‐linking bath containing 2% acetic acid (Figure 4), which could indicate that the calcium ions released from the carbonate contributed to the internal gelation of the alginate chains.

### 3.4 In vitro anthocyanin release

**Figure 5:**
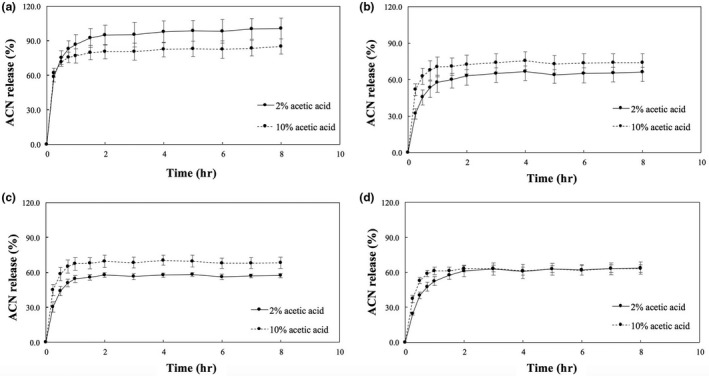
In vitro ACN release profile for (a) control, (b) 1:4, (c) 1:2, and (d) 3:4 CaCO_3_:alginate ratio microspheres prepared with 2 (continuous line) and 10% (dashed line) acetic acid in 0.1 N HCl with surfactant. Bars represent mean ± standard deviation (*n* = 3)

The release profiles of the control and floating microspheres in enzyme‐free simulated gastric fluid are shown in Figure 5. In general, all the samples exhibited a burst phase (up to 15 min) with high amounts of ACNs released into the acidic fluid, followed by a reduction in release rate. For both gelation media, increased concentrations of CaCO_3_ resulted in lower concentrations of ACNs released over time until a plateau was reached (i.e. no more release of the payload).

The Peppas power law equation fitted the release data well (Table 1). All samples exhibited regression coefficient (*R* ^*2*^) values above 91%, small standard errors of regression (*S*), and release exponent values (*n*) below 0.43, indicating that the release of ACNs from the microspheres is controlled most likely by diffusion (Hodsdon, Mitchell, Davies, & Melia, 1995; Peppas, Slaughter, & Kanzelberger, 2012).

**Table 1:** Release kinetics data fitted by the power law, similarity (*f*_*2*_) and difference (*f*_*1*_) factors comparing different CaCO_3_: alginate ratios (1:4, 1:2, and 3:4) and a control (without carbonate)

Sample	2% Acetic acid				10% Acetic acid			
	Control	1:4	1:2	3:4	Control	1:4	1:2	3:4
Power law								
*R* ^*2*^	0.9711	0.9330	0.9191	0.9142	0.9895	0.9723	0.9457	0.9313
*S*	4.93	4.95	4.95	5.85	2.42	3.55	4.73	4.91
*K*	0.82	0.53	0.49	0.48	0.75	0.66	0.62	0.55
*n*	0.12	0.14	0.11	0.17	0.07	0.07	0.07	0.08
Reference								
2% acetic acid								
Control	**––**	25.1334.76	21.3541.15	22.6339.09	44.2313.47	33.8622.36	29.8027.30	25.0934.31
1:4	25.1353.29	**––**	59.439.79	67.496.63	34.7733.57	46.3919.01	55.3211.44	69.965.60
1:2	21.3569.92	59.4310.85	**––**	66.158.23	29.5648.07	38.4031.93	45.0123.53	59.7011.62
3:4	22.6364.17	67.497.10	66.157.95	**––**	30.7143.06	39.7227.46	46.0819.35	58.228.00
10% Acetic acid								
Control	44.2315.46	34.7725.14	29.5632.46	30.7130.10	**––**	52.8910.90	43.8616.57	35.4724.62
1:4	33.8628.80	46.3915.97	38.4024.20	39.7221.54	52.8912.24	**––**	66.046.36	48.0015.39
1:2	29.8037.56	55.3210.26	45.0119.05	46.0816.21	43.8619.87	66.046.80	**––**	59.349.64
3:4	25.0952.23	69.965.56	59.7010.41	58.227.42	35.4732.66	48.0018.19	59.3410.67	**––**

*R* ^*2*^, regression coefficient; *S*, standard error of the regression; *k*, kinetic constant; *n*–release exponent; *f*_*2*_ and *f*_*1*_ are indicated in the first and second lines of each cell, respectively.

A comparison of the similarity and difference factors is shown in Table 1. The release curves showed a difference ≤10% for the following pairs: [2%_1:4, 2%_1:2], [2%_1:4, 2%_3:4], [2%_1:2, 2%_3:4], [10%_1:4, 10%_1:2], [10%_1:2, 10%_3:4], [2%_1:4, 10%_3:4], and [2%_3:4, 10%_3:4].

## 4 Discussion

Researchers have shown that the carriers involved in ACN uptake in vivo can be saturated with the administration of high concentrations of these compounds, with a considerable reduction in the amount absorbed (Kurilich, Clevidence, Britz, Simon, & Novotny, 2005; Talavéra et al., 2003). This is the first time that floating microspheres are proposed as potential GRS for the delivery of ACNs. It is expected that the floating microspheres should be able to sustain the release of ACNs over time to prevent this saturation and overcome losses due to gastric emptying.

Alginate gels have been commonly formed by ionotropic gelation with divalent calcium ions (Mørch, Donati, Strand, & Skjåk‐Bræk, 2006), by either internal or external cross‐linking (Chan, Lee, & Heng, 2006). External gelation has been preferred for encapsulation purposes as it generally produces particles with smoother surface and greater strength and stiffness (Chan et al., 2006). This method has been used for the encapsulation of cocoa extract (Lupo, Maestro, Gutiérrez, & González, 2015), pomegranate peel extract (Zam, Bashour, Abdelwahed, & Khayata, 2014), thyme aqueous extract (Stojanovic et al., 2012), among others. In this study, CaCO_3_ was used as the gas‐generating compound for the preparation of floating microspheres, where upon extrusion of the alginate solution into an acidic gelation medium, the insoluble calcium carbonate reacts with acetic acid to produce CO_2_ gas. Two concentrations of glacial acetic acid were investigated in this study (2 and 10%, v/v) to determine which was more effective in generating gas from the carbonate.

In general, the size of fresh particles reduced by half after drying. The size of dry particles was significantly affected by the concentration of CaCO_3_ in comparison with control samples, corroborating the results obtained by Choi et al. (2002) (from 1.10 ± 0.04 to 1.87 ± 0.05 mm of control particles and the ones prepared with a 3:4 ratio, respectively). In addition, the size was also affected by the concentration of acetic acid used in the gelation medium, that is increasing the concentration of acid (10%) resulted in significantly (*p *<* *.05) larger particles, possibly because the generation of gas was more efficient than at 2%.

The floating capacity provides further evidence that the higher concentration of acid released more gas. With the exception of the control, a significantly higher floating capacity was observed for the samples prepared with 10% acetic acid. For these samples, it is interesting to note that the floating capacity of dried particles decreased with the increase in CaCO_3_ concentration. In this study, it was observed that during preparation (fresh particles), increasing the concentration of CaCO_3_ resulted in nearly 100% floating capacity as more gas was produced (Ma et al., 2008), which suggests that gas could have permeated out of the microspheres during drying. Choi et al. (2002) also observed that fresh particles have a different floating capacity than dry ones as they can contain more gas.

The acid concentration in the gelation bath also impacted the EE. A lower EE was found for control samples prepared with 10% than with 2% acetic acid. The more acidic conditions found in the gelation medium containing 10% acetic acid could have led to a rapid precipitation of alginate as alginic acid, possibly resulting in a more porous shell than the one produced with lower concentration of acid (Hodsdon et al., 1995). The EE results reported in this study are lower than previously described by Nayak, Jain, and Pandey (2011) (77.61–82.48%), Shishu et al. (2007) (71.85–86.45%), and Singh, Deol, and Kaur (2012) (74.37–75.0%), however significantly higher than those reported by Ma et al. (2008) (19.49–20.12%). For floating microspheres, it is possible that the calcium ions released from the salt could have contributed to the internal gelation process as higher EE were observed at higher concentrations of carbonate.

In relation to release, Choi et al. showed that increased concentrations of CaCO_3_ resulted in prolonged release of riboflavin from alginate particles, possibly due to this internal gelation promoted by calcium ions. For samples prepared in both gelation media, increasing the concentration of carbonate resulted in lower amounts of ACN released. The Peppas power law equation was a good fit for the experimental data, with *R* ^*2*^ ranging from 91.91 to 98.95%. The release exponent (*n*) below 0.43 suggested that the release was most likely controlled by diffusion (Hodsdon et al., 1995; Peppas et al., 2012), in agreement with Singh, Sharma, and Chauhan (2010)and Zhang et al. (2011).

The release curves were compared using the similarity (*f*_*2*_) and difference (*f*_*1*_) factors as a measure of agreement between them. The *f*_*2*_ factor varies from 0 to 100 (Shah, Tsong, Sathe, & Liu, 1998), with higher values indicating more similarity. In general, *f*_*2*_ values between 50 and 100 indicate a difference (*f*_*1*_) between the profiles that is equal or smaller than 10%. Considering the release curves that showed a difference ≤10%, particles with a 1:4 ratio prepared with 2% acetic acid exhibited a similar profile to particles with the other concentrations of carbonate. In the case of experiments using the 10% acetic acid, the release curve for the particles with a 1:4 ratio was similar to the curve for the 1:2 ratio, whereas the curve for the 1:2 ratio was similar to that for the 3:4 ratio.

Taking all of the data into consideration, the sample prepared with 10% acetic acid and 1:4 CaCO_3_:alginate ratio displayed the best results in comparison to the other samples. Although the size and EE was slightly lower than at 1:2 and 3:4 ratios, the floatability of dry particles was better, possibly because higher concentrations of carbonate could have burst the microspheres during gelation.

## 5 Conclusion

In this study, floating alginate microspheres were prepared by ionotropic gelation method for the oral delivery of ACNs extracted from haskap berries, using CaCO_3_ as gas‐generating compound. It was found that the inclusion of carbonate resulted in larger particles, higher EE, and lower concentrations of ACNs released over time. The floatation capacity of the microspheres was affected by the concentration of acetic acid used in the gelation media, that is, higher concentration improved floatation. The Peppas power law equation fitted the data well, suggesting that the main mechanism of release was most likely diffusion. Floating microspheres produced with 10% acetic acid could be used as a GRS for the delivery of ACNs in their absorption window, especially with a 1:4 CaCO_3_:alginate ratio. Further studies should optimize the encapsulation method and characterize the microsphere structure to determine a possible interaction between ACNs and the particle components, as well as the release profile and bioavailability in animal models.

## Conflict of Interest

The authors report no declaration of interest.
